# The effectiveness of Carie-Care™, chemomechanical caries removal technique in primary teeth: randomized controlled clinical trial

**DOI:** 10.1186/s12903-023-03594-8

**Published:** 2023-11-18

**Authors:** Alaa Y. Ghanem, Dalia M. Talaat, Marwa M. Essawy, Niveen Bakry

**Affiliations:** 1 Pediatric Dentistry and Dental Public Health Department, Faculty of Dentistry, Alexandria University, Alexandria, Egypt; 2 Oral Pathology Department, Faculty of Dentistry, Alexandria University, Alexandria, Egypt

**Keywords:** Atraumatic restorative technique, Carie-Care™, Primary teeth

## Abstract

**Background:**

Chemomechanical techniques for caries removal have been introduced to overcome the shortcomings of the conventional rotatory instruments. This study aimed to clinically evaluate the effectiveness of the chemomechanical method of caries removal (Carie-Care™) over mechanical caries removal through the Atraumatic Restorative Technique in pediatric patients.

**Methods:**

A randomized controlled clinical trial was conducted. Fifty children fulfilling inclusion criteria were recruited from the outpatient clinic of Pediatric and Dental Public health department, Faculty of Dentistry, Alexandria University. Fifty open carious primary molars were randomly assigned into 2 equal groups according to the method of caries removal. In Group I (test group), caries was removed using the Carie-Care™ system and in Group II (control group), by using mechanical caries removal through the atraumatic restorative technique. Resin modified glass ionomer was used for teeth restoration, the two techniques were compared in each child according to time taken for caries removal, efficacy of caries removal, pain assessment, and child behavior.

**Results:**

The mean time taken for caries removal in the Carie-Care™ treatment group was (575.6 ± 114.8) seconds which was statistically significant higher as compared with the ART treatment group (346.1 ± 97.4) seconds (*p* < 0.001). The mean score of efficacy in caries removal was (0.6 ± 0.8) in the Carie-Care™ group, and (1.3 ± 0.7) in the ART treatment group. When compared to ART, Carie-Care™ was significantly more efficient in caries removal (*p* < 0.002). When pain was assessed by the SEM scale, it was observed that the Carie-Care™ caries removal technique showed statistically significantly more comfort during the procedure compared with the ART procedure (*p* < 0.001).Moreover, children in the Carie-Care™ group enjoyed the process and showed more cooperative behavior when assessed at the end of procedure than those in the ART group with statistically significant difference (*p* = 0.002).

**Conclusions:**

Removal of carious tissue in primary teeth using Chemomechanical Carie-Care™ gel proved to be more time consuming than ART, but on the other hand it was more efficient, comfortable, and accepted by the pediatric patients.

## Background

Dental caries is the most common childhood disease worldwide. Despite reliable scientific advancements and the fact that caries is preventable, the disease continues to be one of the major public health issues [1].

According to estimates, dental caries affects about 60–90% of school-age children. This percentage fluctuates significantly depending on the population, where the incidence of dental caries being much higher in developing countries like the Middle East than in developed [2].

When dental caries is removed using traditional rotary devices, the pulp is frequently subjected to pressure and heat effects that usually cause pain. In addition, drilling could sometimes involve the removal of sound tooth tissue close to the affected caries area [3, 4]. Alternative minimally invasive techniques such as ART and chemo-mechanical caries removal (CMCR) were developed due to the shortcoming of the drill. The common feature of these techniques is the ability to remove only the caries-infected tissue while sparing the caries-affected tissue [5].

The Mechanical caries removal by ART is restoring teeth with hand instruments for decay removal and fluoride releasing adhesive materials (resin modified glass ionomer) for filling. It is the most documented alternative to traditional drilling for dentine caries removal. There are several advantages for the ART method when used in children, such as the elimination of noise and vibration of the handpiece, water coolant and high velocity suction [6].

The CMCR technique has attracted the attention of dental researchers due to its concept of sparing unaffected tooth structure while assuring the removal of the denatured collagen stage of carious dentine [7]. The first CMCR material was developed in the 1970s by the endodontist Goldman, based on n-monochloroglycine and 5% sodium hypochlorite (5% NaOCl) for removal of organic materials in the root canals but presented low effectiveness and slow action [8].

The CMCR agents are either sodium hypochlorite based such as Carisolv or Papain based such as Papacarie [8].Even though they are useful especially in children with dental anxiety, their use in developing countries is limited, due to their expensive cost [9]. Carie-Care™ chemomechanical caries removal material based on papain has been introduced into the market as an economic and cost effective agent [10]. It consists of papaya extract, clove oil, colored gel, chloramines, sodium chloride and sodium methylparaben. Papain, has antibacterial and anti-inflammatory effects. Chloramines aid in the healing process, accelerates tissue repair, and have the ability to dissolve carious dentin by means of chlorination of partially degraded collagen. Clove oil has analgesic and antiseptic effects, whereas sodium methylparaben serves as a preservative. The preparation contains gelling agent in accurate percentage to give exact consistency to the gel to prevent spill over when applied [11].

Few studies were conducted to study the effectiveness of Carie-Care™ as a CMCR agent. Kumar et al. in 2016 [12] compared the effectiveness of different caries removal techniques in mandibular primary molars using atraumatic restorative technique (mechanical caries removal), Smart Burs and Carie-Care™ (chemomechanical caries removal) for primary school children in clinical and community-based settings. They found that Carie-Care™ was superior to the mechanical caries removal technique in terms of time, efficacy, and acceptance in both clinical- and community-based settings [12].

Moreover, Sontakke et al. in 2019 [13], compared the clinical efficiency of Carie-Care™ over the traditional drilling method in permanent molars with class I open carious lesions in children aged 12–15 years. They found Carie-Care™ to be more comfortable for all children [13].

Papacarie and the earlier CMCR agents, do not contain essential oils which have been included in Carie-Care™ and serves as an anti-inflammatory agent. In addition to softening infected dentin, Carie-Care™ has anti-inflammatory and mild anesthetic effects. Therefore complete patient co-operation is not required [14].

Clinical studies on the Carie-Care™ in primary teeth are scarce in the literature [12, 13]. Hence, the aim of this study was to evaluate the efficiency of the Carie-Care™ CMCR agent versus ART in terms of the time taken and efficacy for caries removal, together with child pain perception and behavior. Other factors that may affect the assessment parameters of this study, such as gender and previous dental visits, were also investigated.

The tested null hypothesis stated that there was no significant difference between CMCR method and mechanical caries removal by ART in terms of the time taken for caries removal, efficacy of caries removal, pain assessment, and child behavior.

## Methods

### Cytotoxicity assay

Before the clinical application of the Carie-Care™ gel, its cytotoxicity was assessed in human gingival fibroblasts isolated from patients who underwent clinical crown lengthening, and signed informed consent was obtained from all participants. At a cell density of 7 × 103/well, gingival fibroblasts from passages to 4–6 were seeded onto a 96-well plate and incubated for 24 h in 5% CO^2^ at 37 °C. The cells were then treated with a serial concentration of (0.01-10% v/v gel/growth medium). After a 24 h treatment interval, the cytotoxicity assay was performed according to the well-documented protocol of the MTT-dimethyl sulfoxide assay [15, 16].

### Study design

This study was a randomized controlled clinical trial in which two methods of caries removal were compared following the consort guidelines [17]. The study was approved by the Research Ethical Committee of the Faculty of Dentistry, Alexandria University (#IRB NO:0312 − 10/2021), and registered at the National Institutes of Health (No. NCT05538559) on (14/09/2022).

### Sample size estimation

The sample size was based on a 95% confidence level to detect differences in caries removal time between the mechanical and chemo-mechanical removal methods. Pascareli-Carlos et al. [18] reported mean ± SD caries removal time = 9.20 ± 2.31 min in case of chemo-mechanical caries removal, and 8.40 ± 2.62 in case of the mechanical method (ART). The calculated mean ± SD difference = 0.8 ± 2.46, 95% confidence interval= -0.78, 2.38. The required sample size was calculated to be 22 per group, which increased to 24 to compensate for cases lost to follow-up. The total sample size required was = number of groups × number per group = 2 × 24 = 48 [19].

### Patient recruitment

Two hundred and fifty children were assessed for eligibility at the outpatient clinics of the Pediatric Dentistry and Dental Public Health Department, Faculty of Dentistry, Alexandria University, from March 2022 to November 2022.Two hundred children with radiographic findings of pulp furcation, periapical pathosis and proximal caries evidenced on bitewing radiographs were excluded from the study, and 50 children of both genders aged 5–8 years who met the inclusion criteria were enrolled in the study. Children selected for this study were those with open carious lesions with dentine involvement, but did not reach the pulp (scores five or six, according to the International Caries Detection and Assessment System [ICDAS] [20].The carious lesion had to be wide enough to allow the entrance of the excavator and a behavior rating of 3 or 4 according to Frankl et al. [21].

Informed consent from the parents and/or legal guardians, as well as the children’s acceptance of participating in the study, were obtained. Patients and their parents were informed that the procedure would be videotaped to document their level of comfort.

### Randomization technique, grouping, and allocation concealment

Fifty children were randomly assigned to two groups according to the caries removal method. Each group comprised 25 carious primary molars; Group I(test group), caries removal by Carie-Care™ chemo-mechanical agent, and Group II(control group), caries removal by the ART method. Randomization was done by the permuted block randomization technique, and the allocation sequence was generated using random allocation software where participants were allotted in blocks of 4. The allocation sequence was then sealed in opaque envelopes by a trial-independent individual who was responsible for keeping the envelopes and unfolding them only at the time of treatment. Blinding of the operator and participants could not be performed because of the difference between the two techniques, but the statistician was blinded to the type of treatment [22, 23].

### Examiner reliability

The main researcher, a pediatric dentist, was trained and calibrated to record caries removal efficacy by Munshi et al. [24], pain assessment using the SEM scale [25], and child behavior assessment by Frankl et al. [21]. To set the researcher for the aforementioned assessments, a pilot study was conducted on 20% of the sample that was not included in the study. The kappa statistic was calculated to ensure a high degree of intra-examiner reliability [26]. Intraexaminer reproducibility revealed excellent intraclass correlation coefficients (ICCs) (0.90) and Cohen’s kappa coefficients (K) (0.82).

### Intervention

**Fig. 1:**
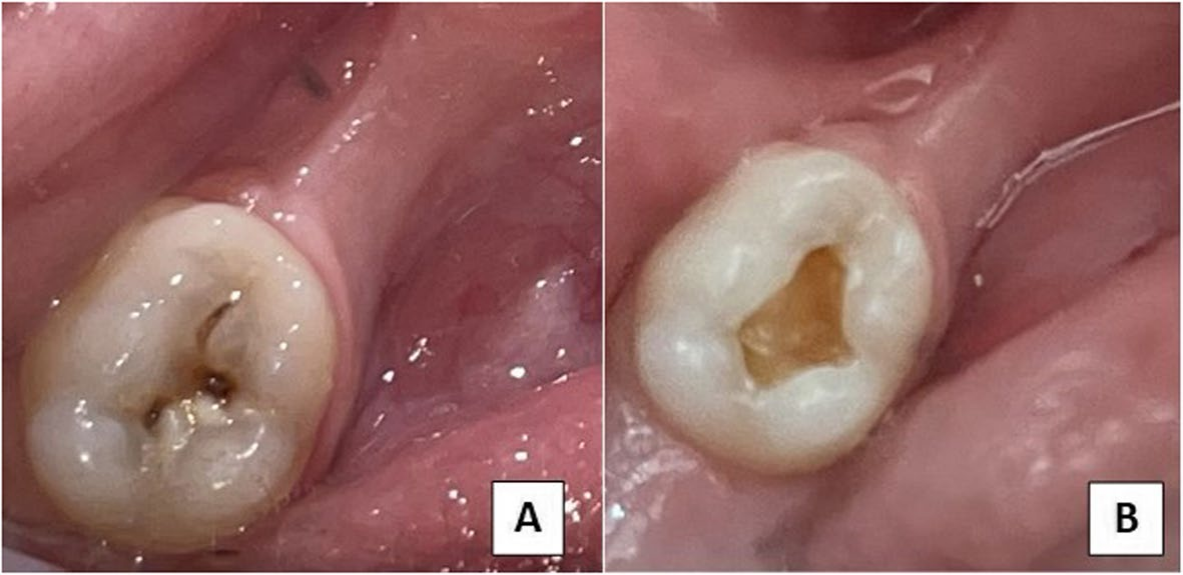
**A** Carious lower right E before Carie-Care^TM^ application. **B** Lower right E after caries removal using Carie-Care^TM^

For both groups, the selected teeth were partially isolated using cotton rolls and saliva ejectors, and caries was removed using one of the following two methods: for. Group I(test group),caries was removed using Carie-Care™ (Uni-biotech Pharmaceuticals PrivateLimited, India),according to the manufacturer’s instructions. The carious cavity was filled with Carie-Care™ gel for 60 s. When the gel had a cloudy appearance, the softened dentin was scraped away using a sterile spoon excavator (Nordent Manufacturing Inc. USA) without applying pressure. It was reapplied until its light color remained unchanged, and the cavity surface felt hard on excavation and was considered the endpoint. Residual gel was removed using moistened cotton pellets [12] (Fig. 1A, B). In Group II (control group), caries was removed by ART. An enamel hatchet (Nordent Manufacturing Inc. USA) was used to widen the lesion to remove unsupported enamel rods and then caries was removed using a spoon excavator [12]. Caries excavation end point was detected when firm dentin (resistant to hard excavation) was reached [27, 28]. All teeth included in the study were restored using a resin- modified glass ionomer (SDI Riva Light Cure ,SDI Limited, Australia).

### Assessment procedures

The following was measured for each patient:

The time taken for the two techniques was measured from the start of the caries removal procedure until the caries excavation endpoint by using a stopwatch and was recorded in seconds.

 Efficacy of caries removal: Complete caries excavation was verified by the tip of a blunt explorer (Nordent Manufacturing Inc. USA), which moved smoothly across the dentinal wall and did not give a tug-back sensation [28]. Caries removal was rated according to Munshi et al.’s [24] scoring criteria (Table 1).

**Table 1:** Numerical scoring criteria for caries removal status

Score	Scoring criteria
0	Caries removed completely
1	Caries present in the base of the cavity
2	Caries present in the base and/or one wall
3	Caries present in the base and/or two walls
4	Caries present in the base and/or more than two walls
5	Caries present in the base, walls and margins of the cavity

Child pain perception to the respective procedure during caries removal was assessed using the Sound, Eye, and Motor (SEM) scale according to Wright et al. [25], using the recorded videotape. This objective method of pain assessment (patient comfort level) measured patient comfort based on three types of observations shown by the patient: sound, eye, and motor. A numerical value (score) was given to the type of response obtained for each observation, where 1 = comfort, 2 = mild discomfort, 3 = moderately painful, and 4 = painful. Mean values were calculated to determine the comfort level.

Child behavior was evaluated immediately upon completing the procedure according to the behavior categories of Frankl et al. [21].

### Statistical analysis

IBM SPSS software package version 20.0. (IBM Corp., Armonk, NY,) was used for the data analysis. Qualitative data are represented as numbers and percentages, and the Chi-square test was used to compare the two study groups. Quantitative continuous data were tested for normality using the Shapiro-Wilk test and expressed as range (minimum and maximum), mean, standard deviation, and median. When quantitative variables were not normally distributed, the Mann-Whitney test was applied to identify intergroup differences. The results were judged at a 5% level of significance [29].

Statistical analysis showed that the half-maximal inhibitory concentration (IC50) of the Carie-Care™ gel was analyzed by a non-linear regression test using GraphPad Prism 8.0.1.

## Results

**Fig. 2:**
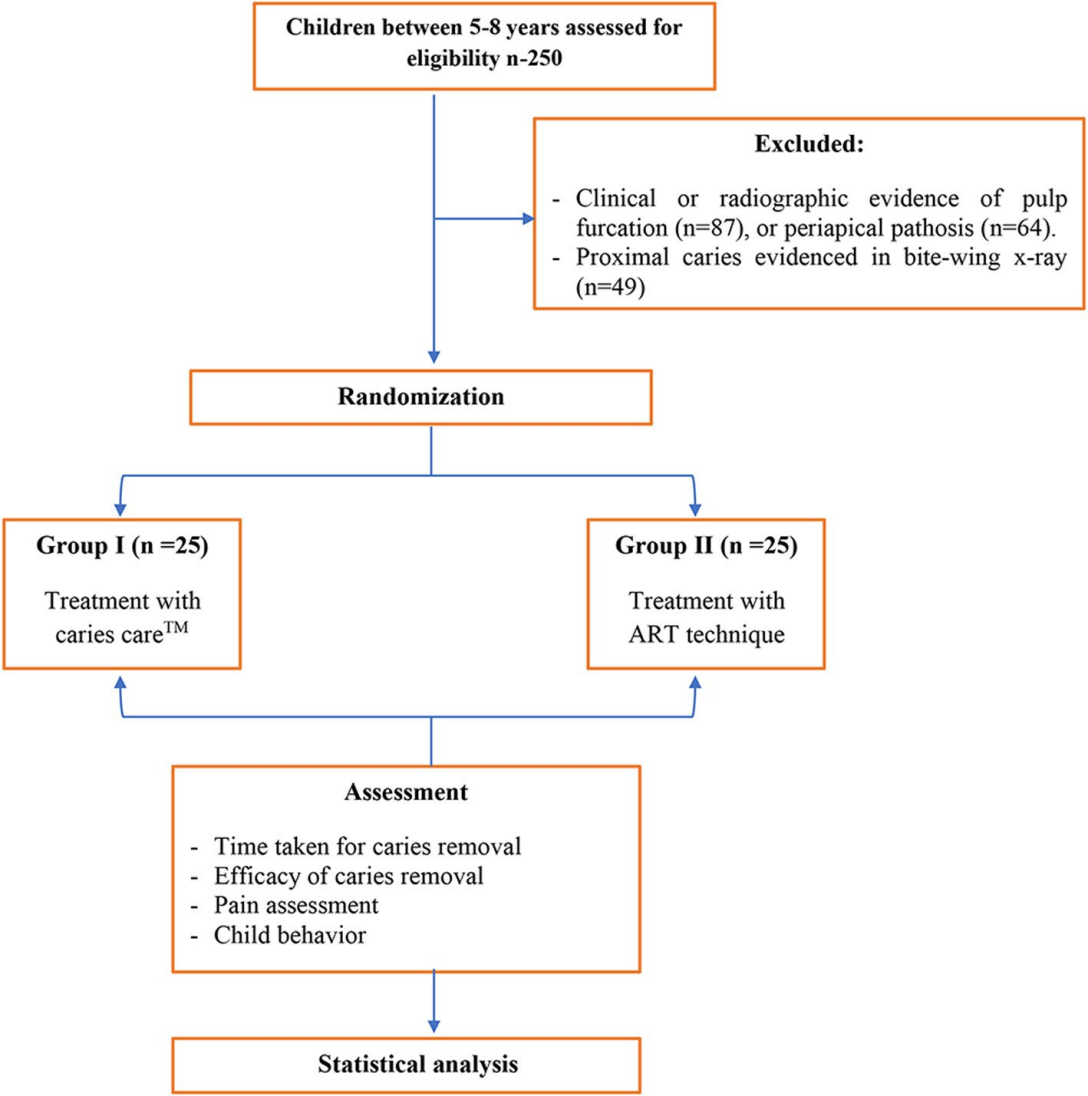
CONSORT flow diagram of the study design

 Two hundred and fifty children were examined of which fifty children met the inclusion criteria and were enrolled in the study (Fig. 2). Children were aged 5–8 years with a mean age of 6 years in the Carie-Care™ treatment group (group I) and 5.9 years in the ART treatment group (group II). Boys constituted 48%and 44% of groups I and II, respectively. Regarding the history of previous dental visits, 40% of the children in group I and 36% of the children in group II had previous dental visits. Differences in age, sex, and history of previous dental visits between the two study groups were not statistically significant. (*p* = 0.360, 0.776, and 0.771, respectively) (Table 2).

**Table 2:** Distribution of children in the two study groups according to personal data and previous dental visit

	Carie-Care™ (*n* = 25)	ART (*n* = 25)	Test of Sig.	*P*
**Age (years)**				
Mean ± SD.	6.1 ± 0.8	5.9 ± 0.8	U = 268.50	0.360
Median (Min. – Max.)	6 (5–8)	5.9 (5–8)		
**Gender**				
Boys	12 (48%)	11 (44%)	χ^2^ = 0.081	0.776
Girls	13 (52%)	14 (56%)		
**Previous dental visit**				
No	15 (60%)	16 (64%)	χ^2^=0.085	0.771
Yes	10 (40%)	9 (36%)		

*SD* Standard deviation, *U* Mann Whitney test, *X *^2^ Chi square test, *p* *p* value for comparing between the two studied groups

**Fig. 3:**
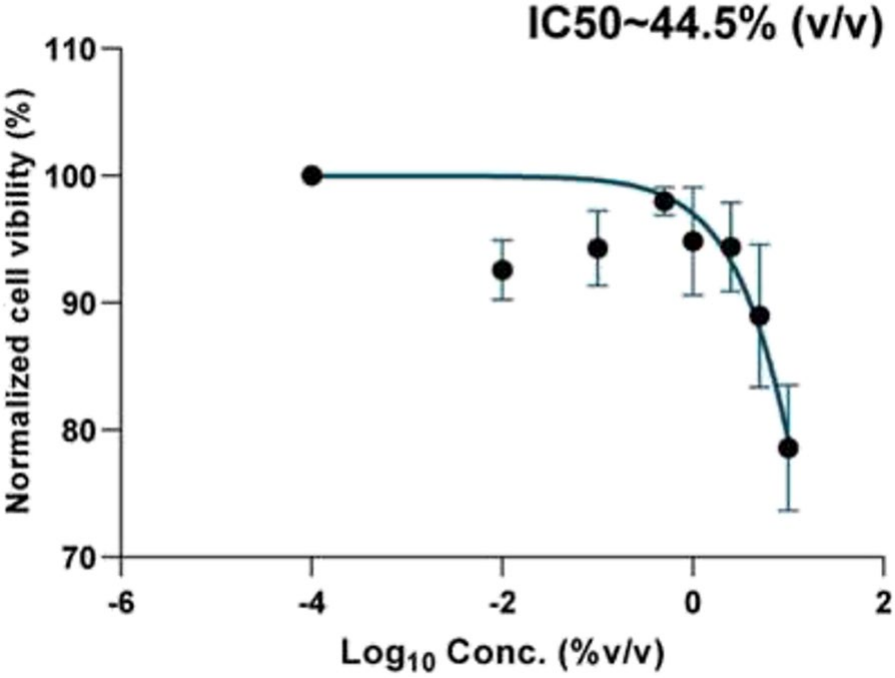
The dose-dependent curve of the safety profile of gel on human gingival fibroblasts, where the gel records its half-maximal inhibitory dose at ~ 44.5% of the applied volume. The data is the mean ± SD of triplicates in three independent experiments

 Before the clinical application of Carie-Care™, its cytotoxicity was assessed in human gingival fibroblasts, where it was safe in a dose-dependent manner. The cytotoxicity increased with increasing gel concentration with a safety IC50 up to 44.5% (v/v), which was far above the concentration used. Moreover, the gel consistency prevented the spillage of the applied amount to flow easily and contact the child’s mucosa (Fig. 3).

The mean time for complete caries removal using Carie-Care™ was longer (575.6 ± 114.8 s) than that with ART (346.1 ± 97.4 s). The difference was statistically significant at *p* < 0.001.

The mean score for the efficacy of caries removal according to the Munshi et al. scoring criteria showed a statistically significant intergroup difference in favor of the Carie-Care™ group (*p* = 0.002).

 Pain assessment by the SEM scale showed that all of the children (100%) in the Carie-Care™ treatment group demonstrated comfort (score 1). However, in the ART group, only 60% scored 1, and 40% demonstrated mild discomfort (score 2).Comparing the two groups, children treated with the Carie-Care™ removal technique showed more comfort during the procedure than the ART with statistical significance (*p* < 0.001). Moreover, children in the Carie-Care™ group were significantly more cooperative and enjoyed the situation than those in the ART group (*p* = 0.002) (Table 3).

**Table 3:** Comparison of assessment parameters in the study groups

	Carie-Care™ (*n* = 25)	ART (*n* = 25)	Test of Sig.	*P*
**Time (/sec)**				
Mean ± SD.	575.6 ± 114.8	346.1 ± 97.4	U = 46.500^*^	< 0.001^*^
Median (Min. – Max.)	605 (360–720)	360 (180–480)		
**Behavior**				
Mean ± SD.	3.7 ± 0.5	3.2 ± 0.4	U = 175.0^*^	0.002^*^
Median (Min. – Max.)	4 (3–4)	3 (3–4)		
**Efficacy of caries removal**				
Mean ± SD.	0.6 ± 0.8	1.3 ± 0.7	U = 165.50^*^	0.002^*^
Median (Min. – Max.)	0 (0–2)	1 (0–2)		
**SEM scale**				
1	25 (100%)	15 (60%)	χ^2^=12.500^*^	< 0.001^*^
2	0 (0%)	10 (40%)		

*SD* Standard deviation, *U* Mann Whitney test

*: Statistically significant at *p* ≤ 0.05

 When the assessment parameters were compared between boys and girls in the two study groups, statistically significant differences were not observed in terms of the time taken for caries removal, efficacy of caries removal, and child behavior during the procedure (*p* = 0.894, 0.470, and 0.57 respectively) (Table 4).

**Table 4:** Relation between gender and assessment parameters in the study groups

		**Boys**	**Girls**	**U**	**P**
**Carie-Care**^TM^** (*n* = 25)**	**Time (/sec)**	**(*n* = 12)**	**(*n* = 13)**		
	Mean ± SD.	604.6 ± 99.5	548.8 ± 125.2	63.50	0.437
	Median (Min. – Max.)	607.5 (420 – 720)	545 (360 – 690)		
	**Behavior**	**(*n* = 12)**	**(*n* = 13)**		
	Mean ± SD.	3.7 ± 0.5	3.7 ± 0.5	76.00	0.936
	Median (Min. – Max.)	4 (3 – 4)	4 (3 – 4)		
	**Efficacy of caries removal**	**(*n* = 12)**	**(*n* = 13)**		
	Mean ± SD.	0.3 ± 0.5	0.9 ± 0.9	48.00	0.110
	Median (Min. – Max.)	0 (0 – 1)	1 (0 – 2)		
**ART (*n* = 25)**	**Time (/sec)**	**(*n* = 12)**	**(*n* = 13)**		
	Mean ± SD.	350.8 ± 94.4	341.8 ± 103.8	75.50	0.894
	Median (Min. – Max.)	365 (180 – 450)	360 (180 – 480)		
	**Behavior**	**(*n* = 12)**	**(*n* = 13)**		
	Mean ± SD.	3.2 ± 0.4	3.3 ± 0.5	67.00	0.574
	Median (Min. – Max.)	3 (3 – 4)	3 (3 – 4)		
	**Efficacy of caries removal**	**(*n* = 12)**	**(*n* = 13)**		
	Mean ± SD.	1.2 ± 0.8	1.5 ± 0.5	64.00	0.470
	Median (Min. – Max.)	1 (0 – 2)	1 (1 – 2)		

*SD* Standard deviation, *U* Mann Whitney test

 Children in the Carie-Care™ group who did not experience a previous dental visit had a significantly better behavior rating score and efficacy caries removal score than those who had a previous dental visit (*p* < 0.001, and 0.007 respectively). However, whether or not a child experienced a previous dental visit had no effect on the time taken for caries removal (*p* = 0.216). In the ART group, no significant association was observed between the presence of a previous dental visit and assessment parameters (Table 5).

**Table 5:** Relation between previous dental visit and assessment parameters in the study groups

		Previous dental visit		U	P
		No (*n* = 15)	Yes *(n = 10)*		
**Carie-Care™(*n* = 25)**	Time (/sec)				
	Mean ± SD.	596.5 ± 98.3	544.3 ± 135.3	52.50	0.216
	Median (Min. – Max.)	610 (447–720)	600 (360–690)		
	**Behavior**				
	Mean ± SD.	4 ± 0	3.2 ± 0.4	15.00^*^	< 0.001^*^
	Median (Min. – Max.)	4 (4–4)	3 (3–4)		
	**Efficacy of caries removal**				
	Mean ± SD.	0.3 ± 0.5	1.2 ± 0.8	27.00^*^	0.007^*^
	Median (Min. – Max.)	0 (0–1)	1 (0–2)		
**ART (*n* = 25)**	**Time (/sec)**	**(*n* = 16)**	**(*n* = 9)**		
	Mean ± SD.	352.5 ± 83.1	334.8 ± 123.8	69.50	0.890
	Median (Min. – Max.)	360 (180–450)	370 (180–480)		
	**Behavior**				
	Mean ± SD.	3.3 ± 0.5	3.1 ± 0.3	57.50	0.419
	Median (Min. – Max.)	3 (3–4)	3 (3–4)		
	**Efficacy of caries removal**				
	Mean ± SD.	1.4 ± 0.8	1.1 ± 0.3	47.00	0.169
	Median (Min. – Max.)	2 (0–2)	1 (1–2)		

*SD* Standard deviation, *U* Mann Whitney test

*: Statistically significant at* p *≤ 0.05

## Discussion

The present randomized controlled clinical study aimed to evaluate the efficiency of Carie-Care™ as a chemo-mechanical caries removal agent and compare it with that of ART. To the best of authors’ knowledge, the present study is the first in Egypt and the Easternmediteranean/North African region to evaluate the effectiveness of Carie-Care™ CMCR agent versus ART in pediatric patients in terms of time taken and efficacy for caries removal, together with child pain perception and behavior. To fulfill the aims of this study, fifty children of both genders aged 5–8 years who fulfilled the inclusion criteria were selected from the outpatient clinic. Fifty open carious primary molars were randomly assigned to two equal groups according to the caries removal method. In this study the split mouth technique was not used because of the difficulty to find carious lesions symmetrically distributed in the mouth of children. Based on the findings of the current study, the null hypothesis was rejected. A significant difference was detected between the two procedures in terms of the time required, efficacy of caries removal, pain assessment, and child behavior.

The results of the present study revealed that the mean time taken to completely remove caries in the Carie-Care™ treatment group was significantly longer than that in the ART treatment group. This finding could be due to the repeated application of the gel to completely remove caries [30]. These results are in accordance with those of several studies [31–36]. Moreover, Soni et al. reported that significantly more time was required to remove caries from teeth treated with chemo-mechanical techniques than from those treated with hand instruments [37]. Similar results were reported in other studies [38–40].

There is continuous debate over the most effective strategy for treating caries in primary teeth, especially for multisurface lesions. In the present study, although Carie-Care™ was time consuming, it was significantly more efficient in caries removal than ART. This finding is in agreement with Kumar et al. [12], who found Carie-Care™ to have the greatest efficacy in both clinical and community-based settings when compared to ART and polymer burs. Sahana et al. [41] reported that Carie-Care™ is efficient and conservative for caries removal. Similar results have been reported by other researchers [32, 35, 42–47].

On the other hand, Banerjee et al. [48] in their study on ART caries removal found that the use of hand excavators was the most suitable technique for carious dentine excavation in deciduous teeth [46]. In addition, Celiberti et al. [49] showed that hand excavators are the most effective instruments for cleaning cavities in primary teeth. However, Nadanovsky et al. reported no difference between cavities treated with a chemo-mechanical caries removal gel and those treated with hand instruments in terms of the amount of caries left behind [50].

Pain assessment in the present study showed significant comfort during the use of Carie-Care™ compared with the ART technique in caries removal. The CMCR gel is effective only on the denuded fibers in demineralized dentine, thus preventing painful removal and damage to healthy dentine. Moreover, the gel itself has a heat-insulating function as it covers the cavity during the procedure, and thus reduces pulpal stimulation. In addition, during ART caries removal, the unpleasant feeling of scraping the decay with hand excavation makes the procedure more stressful than the chemomechanical caries removal method [10].

In agreement with the results of the present study, Soni et al. [37] and Pandit et al. [39] found that the CMCR agent was less painful than a spoon excavator. This was in agreement with several studies, which found that the enzyme-based agents, such as Carie-Care™, were reported to cause significantly less pain than all other methods of caries removal [13, 51–53].

Child behavior was evaluated in the current study immediately upon completion of the procedure, showing that children in the Carie-Care™ group were significantly more cooperative and enjoyed the procedure than those in the ART group. The absence of pain in the CMCR treatment group was the cause of the child cooperative behavior. In agreement with the results of Kumar et al. [12], Carie-Care™ was the most acceptable method for caries removal, followed by ART. In addition, Nadanovsky et al. [50] used the verbal pain scale to report the discomfort experienced by patients during caries removal by a CMCR agent and hand instruments, and discovered that most patients found the chemo-mechanical method to be more comfortable. In addition, a review article by Venkataraghavan et al. [10] concluded that Carie-Care™ can be an alternative in many cases, especially in children who are usually anxious about dental procedures.

The results of the current study showed no significant difference between boys and girls regarding assessment parameters (time taken for caries removal, efficacy of caries removal, and child behavior) during the procedure. On the contrary other studies found that dental procedures were affected by the child gender where boys were less dentally anxious than girls which goes with the nature of boys who prefer to seem strong. [54, 55]. However, Sharma et al. [56] found that girls were more cooperative and responsive than boys, which was attributed to the quieter nature of girls.

Studying the influence of previous dental visit experience on assessment parameters, children in the Carie-Care™ treatment group who did not experience a previous dental visit had a significantly better behavior rating score and efficacy of caries removal score than those who had a previous dental visit, which could be related to a history of dental pain from invasive procedures performed in previous dental visits. This is consistent with previous studies [56, 57]. However, in the ART group, no significant association was found, which may be due to the simple form of dental procedures that these children underwent during their previous visits. A limitation of the present study was the recruitment of children with carious primary molars, based on the inclusion criteria. Another limitation is that another blinded evaluator was not recruited for this study. In addition, no uncooperative patients were included. Therefore, further studies on pediatric patients with various levels of cooperation and different age groups are recommended.

Within the limitations of the present study and based on the results, the Carie-Care™ CMCR agent can be recommended as an alternative to ART in pediatric patients, considering its effectiveness in caries removal, patient comfort, and relatively low price, which could be of great benefit, especially for low-income developing countries in Africa. However, future large-scale multicenter trials are recommended to verify the findings of the present study.

## Conclusions

The use of Chemo-mechanical Carie-Care™ gel to remove carious tissue in primary teeth proved to be more time consuming than ART, but on the other hand it was more efficient, comfortable and accepted by the pediatric patients. Authors believe that this study will make useful contributions to the literature and clinicians.

## Data Availability

Patients’ records at the Pediatric Dentistry Department, Faculty of Dentistry, Alexandria University, contain the datasets created and analyzed for the current study and were used under license for this study. The public accessibility of such data is subject to restrictions. However, data from the corresponding author are available upon reasonable request and with permission from the Pediatric Dentistry Department, Faculty of Dentistry, University of Alexandria.

## Abbreviations

ART: Atraumatic restorative technique
CMCR: Chemo-mechanical caries removal

## References

[1] Global strategy on oral health. In (2022). Seventy-fifth World Health Assembly. Provisional agenda item 14.1.

[2] Oral health. In (2021). Seventy-fourth World Health Assembly. Summary and verbatim records.

[3] Kathuria V, Ankola AV, Hebbal M, Mocherla M (2013). Carisolv- an innovative method of caries removal. J Clin Diagn Res.

[4] Kotb RM, Elkateb MA, Ahmed AM, Kawana KY, El Meligy OA (2016). Dentin Topographic Features following chemomechanical caries removal in primary teeth. J Clin Pediatr Dent.

[5] Ganesh M, Parikh D (2011). Chemomechanical caries removal (CMCR) agents: review and clinical application in primary teeth. J Dent Oral Hyg.

[6] Waggoner WF. Restorative dentistry for the primary dentition. In: Nowak AJ, Christensen JR, Mabry TR, Townsend JA, Wells MH, editors. Pediatric dentistry; Infancy through adolescence. 6th ed. Ch 22. Philadelphia, PA: Elsevier; 2019.

[7] Pai VS, Nadig RR, Jagadeesh T, Usha G, Karthik J, Sridhara K (2009). Chemical analysis of dentin surfaces after Carisolv treatment. J Conserv Dent.

[8] Goldman M, Kronman JH (1976). A preliminary report on a chemomechanical means of removing caries. J Am Dent Assoc.

[9] Chowdhry S, Saha S, Samadi F, Jaiswal JN, Garg A, Chowdhry P (2015). Recent vs conventional methods of Caries removal: a comparative in vivo study in Pediatric patients. Int J Clin Pediatr Dent.

[10] Venkataraghavan K, Kush A, Lakshminarayana C, Diwakar L, Ravikumar P, Patil S (2013). Chemomechanical caries removal: a Review & Study of an Indigen-ously developed Agent (Carie Care (TM) gel) in children. J Int Oral Health.

[11] Puri A, Gaurav K, Kaur J, Sethi D, Jindal L, Jain S (2020). Chemomechanical caries removal: an overview. IDA Lud J-le Dent.

[12] Kumar K, Prasad MG, Sandeep RV, Reddy SP, Divya D, Pratyusha K (2016). Chemomechanical caries removal method versus mechanical caries removal methods in clinical and community-based setting: a comparative in vivo study. Eur J Dent.

[13] Sontakke P, Jain P, Patil AD, Biswas G, Yadav P, Makkar DK (2019). A comparative study of the clinical efficiency of chemomechanical caries removal using Carie-Care gel for permanent teeth of children of age group of 12–15 years with that of conventional drilling method: a randomized controlled trial. Dent Res J (Isfahan).

[14] Prabhakar A, Lokeshwari M, Naik SV, Yavagal C (2018). Efficacy of Caries removal by Carie-Care and Erbium-doped Yttrium Aluminum Garnet laser in primary molars: a scanning Electron microscope study. Int J Clin Pediatr Dent.

[15] Ammar N, El-Tekeya MM, Essa S, Essawy MM, El Achy SN, Talaat DM (2022). The antibacterial effect of nanosilver fluoride in relation to caries activity in primary teeth: a protocol for a randomized controlled clinical trial. Trials.

[16] Ammar N, El-Tekeya MM, Essa S, Essawy MM, Talaat DM (2022). Antibacterial effect and impact on caries activity of nanosilver fluoride and silver diamine fluoride in dentin caries of primary teeth: a randomized controlled clinical trial. BMC Oral Health.

[17] Schulz KF, Altman DG, Moher D (2010). CONSORT 2010 Statement: updated guidelines for reporting parallel group randomised trials. BMC Med.

[18] Pascareli-Carlos AM, Martins LF, Silva Gonçalves MD, Pettorossi Imparato JC, Tedesco TK (2021). Pain perception of children after restorative treatments: atraumatic restorative treatment versus chemomechanical removal - A noninferiority randomized clinical trial. J Indian Soc Pedod Prev Dent.

[19] Petrie A, Sabin C (2009). Medical statistics at a glance.

[20] Gugnani N, Pandit IK, Srivastava N, Gupta M, Sharma M (2011). International Caries Detection and Assessment System (ICDAS): a New Concept. Int J Clin Pediatr Dent.

[21] Frankl S (1962). Should the parent remain with the child in the dental operatory?. J Dent Child.

[22] Schulz KF, Grimes DA (2002). Generation of allocation sequences in randomised trials: chance, not choice. Lancet.

[23] Sealed Envelope Ltd. Simple randomisation service. 2021. Available at: https://www.sealedenvelope.com/simple-randomiser/v1/.

[24] Munshi AK, Hegde AM, Shetty PK (2001). Clinical evaluation of Carisolv in the chemico-mechanical removal of carious dentin. J Clin Pediatr Dent.

[25] Wright GZ, Weinberger SJ, Marti R, Plotzke O (1991). The effectiveness of infiltration anesthesia in the mandibular primary molar region. Pediatr Dent.

[26] McHugh ML (2012). Interrater reliability: the kappa statistic. Biochem Med (Zagreb).

[27] Hegde S, Kakti A, Bolar DR, Bhaskar SA (2016). Clinical efficiency of three caries removal systems: Rotary Excavation, Carisolv, and Papacarie. J Dent Child (Chic).

[28] Boob AR, Manjula M, Reddy ER, Srilaxmi N, Rani T (2014). Evaluation of the efficiency and effectiveness of three minimally invasive methods of Caries removal: an in vitro study. Int J Clin Pediatr Dent.

[29] Statistics IS (2014). IBM SPSS statistics software version 22.

[30] Avinash A, Grover SD, Koul M, Nayak MT, Singhvi A, Singh RK (2012). Comparison of mechanical and chemomechanical methods of caries removal in deciduous and permanent teeth: a SEM study. J Indian Soc Pedod Prev Dent.

[31] Katiyar A, Gupta S, Gupta K, Sharma K, Tripathi B, Sharma N (2021). Comparative evaluation of chemo-mechanical and rotary-mechanical methods in removal of caries with respect to Time Consumption and Pain Perception in Pediatrc Dental patients. Int J Clin Pediatr Dent.

[32] Salem RG, Fawzy MI, Mostafa MH (2021). Comparative study of Erbium Laser Versus a chemo-mechanical caries removal method in primary teeth. Al-Azhar D J.

[33] Aswathi KK, Rani SP, Athimuthu A, Prasanna P, Patil P, Deepali KJ (2017). Comparison of efficacy of caries removal using polymer bur and chemomechanical caries removal agent: a clinical and microbiological assessment - an in vivo study. J Indian Soc Pedod Prev Dent.

[34] Bohari MR, Chunawalla YK, Ahmed BM (2012). Clinical evaluation of caries removal in primary teeth using conventional, chemomechanical and laser technique: an in vivo study. J Contemp Dent Pract.

[35] Deng Y, Feng G, Hu B, Kuang Y, Song J (2018). Effects of Papacarie on children with dental caries in primary teeth: a systematic review and meta-analysis. Int J Paediatr Dent.

[36] Jawa D, Singh S, Somani R, Jaidka S, Sirkar K, Jaidka R (2010). Comparative evaluation of the efficacy of chemomechanical caries removal agent (papacarie) and conventional method of caries removal: an in vitro study. J Indian Soc Pedod Prev Dent.

[37] Soni HK, Sharma A, Sood PB (2015). A comparative clinical study of various methods of caries removal in children. Eur Arch Paediatr Dent.

[38] Nagaveni N, Radhika N, Satisha T, Ashwini K, Neni S, Gupta S (2016). Efficacy of new chemomechanical caries removal agent compared with conventional method in primary teeth: an in vivo study. Int J of Oral Health Sci.

[39] Pandit IK, Srivastava N, Gugnani N, Gupta M, Verma L (2007). Various methods of caries removal in children: a comparative clinical study. J Indian Soc Pedod Prev Dent.

[40] Kochhar GK, Srivastava N, Pandit IK, Gugnani N, Gupta M (2011). An evaluation of different caries removal techniques in primary teeth: a comparitive clinical study. J Clin Pediatr Dent.

[41] Sahana S, Vasa AA, Geddam D, Reddy VK, Nalluri S, Velagapudi N (2016). Effectiveness of chemomechanical caries removal agents Papacarie(®) and Carie-Care™ in primary molars: an in vitro study. J Int Soc Prev Community Dent.

[42] Kannaiyan V, Kumar P, Thomas A, Raaje P, Guptha V, Kumar M (2022). Comparative evaluation of caries removal efficacy between chemomechanical agents & conventional methods regarding time consumption: an in vivo study. Int J Health Sci.

[43] Peters MC, Flamenbaum MH, Eboda NN, Feigal RJ, Inglehart MR (2006). Chemomechanical caries removal in children: efficacy and efficiency. J Am Dent Assoc.

[44] Sharma N, Sisodia S, Jain A, Bhargava T, Kumar P, Rana KS (2023). Evaluation of the efficacy of recent caries removal techniques: an in Vitro Study. Cureus.

[45] Kotb RM, Abdella AA, El Kateb MA, Ahmed AM (2009). Clinical evaluation of Papacarie in primary teeth. J Clin Pediatr Dent.

[46] Maashi MS, Elkhodary HM, Alamoudi NM, Bamashmous NO (2023). Chemomechanical caries removal methods: a literature review. Saudi Dent J.

[47] Abdelaziz E, Badran A, Allam G (2022). Chemomechanical caries removal agents and their applications in pediatric dentistry. J Adv Dent Res.

[48] Banerjee A, Watson TF, Kidd EA (2000). Dentine caries excavation: a review of current clinical techniques. Br Dent J.

[49] Celiberti P, Francescut P, Lussi A (2006). Performance of four dentine excavation methods in deciduous teeth. Caries Res.

[50] Nadanovsky P, Cohen Carneiro F, Souza de Mello F (2001). Removal of caries using only hand instruments: a comparison of mechanical and chemo-mechanical methods. Caries Res.

[51] Cardoso M, Coelho A, Lima R, Amaro I, Paula A, Marto CM et al. Efficacy and Patient’s Acceptance of Alternative Methods for Caries Removal-a Systematic Review. J Clin Med 2020;9. 10.3390/jcm9113407 PMC7690910 33114249

[52] Rajakumar S, Mungara J, Joseph E, Philip J, Shilpa Priya MP (2013). Evaluation of three different caries removal techniques in children: a comparative clinical study. J Clin Pediatr Dent.

[53] Hegde AM, Preethi V, Shetty A, Shetty S (2014). Clinical evaluation of chemo-bechanical caries removal using carie-care system among school children. J Health Allied Sci NU.

[54] Gaber AE, Khalil AM, Talaat DMTM (2018). The impact of gender on child dental anxiety in a sample of Egyptian children (a cross-sectional study). Alex Dent J.

[55] Alshuaibi AF, Aldarwish M, Almulhim AN, Lele GS, Sanikommu S, Raghunath RG (2021). Prevalence of Dental fear and anxiety and its triggering factors in the Dental Office among School-going children in Al Ahsa. Int J Clin Pediatr Dent.

[56] Sharma A, Kumar D, Anand A, Mittal V, Singh A, Aggarwal N (2017). Factors predicting Behavior Management problems during initial Dental examination in children aged 2 to 8 years. Int J Clin Pediatr Dent.

[57] Dalal D, Khandelwal A, Tirupathi SP, Tirupathi SP, Kadam N. Identification and Assessment of factors Predicting Behaviour of the child in the First Dental visit. JSPS 2022:10950–4.

